# Pangenome-based association testing between a structural variant located upstream of the *KIT* gene and head depigmentation across a diverse panel of cattle breeds

**DOI:** 10.1186/s12711-026-01037-w

**Published:** 2026-02-26

**Authors:** V. Sorin, M. P. Sanchez, L. Drouilhet, G. Tosser-Klopp, M. M. Naji, D. Boichard, H. Pausch, M. Boussaha, A. S. Leonard

**Affiliations:** 1 Université Paris Saclay, INRAE, AgroParisTech, GABI, 78350 Jouy en Josas, France; 2 Université de Toulouse, INRAE, GenPhySE, 31326 Castanet-Tolosan, France; 3 Animal Genomics, ETH Zurich, 8092 Zurich, Switzerland

## Abstract

Coat color variation is a key phenotypic trait in domestic animals. Among the genetic factors involved, the *KIT* gene has frequently been associated with pigmentation diversity across species. In cattle, spotting or piebald phenotypes have been linked to variation in the genomic region encompassing *KIT*, but the identification of causal variants was not always possible. This is largely due to the regulatory nature of the underlying variants and the structural complexity of this genomic region, which remains difficult to investigate with linear reference genome-based approaches. In the present study, we used a local pangenome strategy to investigate a genomic region on chromosome 6 encompassing *KIT* which was recently suggested to be associated with head depigmentation in white-headed cattle breeds. We constructed a 2 Mb pangenome graph encompassing the associated region using 79 assemblies from 20 cattle breeds. Through the evaluation of the coverage at the node level on this pangenome graph, we identified a ~ 7 kb structural variant which was supported by 21 assemblies only from breeds exhibiting a white-headed phenotype. To validate these findings, we aligned 564 short-read sequencing data to a local graph of 30 kb, spanning the structural variant identified, and computed normalized coverage across the region. White-headed cattle breeds consistently exhibited higher coverage values, while color-headed breeds displayed nearly zero coverage. Together, these results confirm the association between a structural variant upstream of *KIT* with the white-headed phenotype. More broadly, our study demonstrates how targeted local pangenome graphs can efficiently resolve complex structural variants (SVs) with phenotypic impact, offering an interesting and computationally feasible alternative to whole-genome graph approaches.

**Supplementary Information:**

The online version contains supplementary material available at 10.1186/s12711-026-01037-w.

## Background

Variation in coat color is among the most frequently selected phenotypic traits in domestic animals. In addition to its aesthetic and cultural importance, variation in pigmentation can have functional implications, such as influencing thermoregulation [1], camouflage or adaptation to specific environments [2]. In cattle, coat color and spotting patterns often serve as breed identifiers and contribute to breed standards and marketability [3]. Understanding the genetic architecture of pigmentation traits therefore represents not only a question of fundamental biology but also holds practical relevance for animal breeding and conservation.

Among the genes influencing skin pigmentation, *KIT* is of particular interest, as its central role in melanocyte biology translates into characteristic coat color phenotypes that define several breeds. Specifically, this gene encodes a tyrosine kinase receptor essential for the migration, survival and proliferation of melanocytes during embryonic development [4, 5]. The function of *KIT* is highly conserved across vertebrates, and loss-of-function mutations or regulatory alterations often result in pigmentation abnormalities. In several species, including mice, pigs, horses, and cattle, genetic variation nearby the *KIT* gene has been linked to spotting or piebald phenotypes [6–12]. In cattle, various pigmentation traits specific to certain breeds have been linked to the *KIT* gene which is located on bovine chromosome 6 between 70,166,682 bp and 70,254,046 bp (ARS-UCD1.2). For example, a complex structural variant (SV) located in the *KIT* locus has been identified as responsible for the Pinzgauer spotting phenotype [11]. In Holstein, a locus within the *KIT* region, has been reported to be associated with the proportion of black coat color [13]. Similarly, a series of two translocation events—first from chromosome 6 to chromosome 29, and a second one, derived from the first, from chromosome 29 to chromosome 6—of a genomic segment encompassing the *KIT* gene has been linked to color sidedness in Belgian Blue and Brown Swiss [8]. In Fleckvieh and Simmental, head depigmentation has been linked to genetic variants near *KIT* [14, 15]. However, the precise causal mutations have not been identified in these studies. This is primarily attributable to the structural complexity of the *KIT* genomic region, defined here as the upstream and downstream flanking sequences surrounding the gene, which limits the effectiveness of standard approaches based on short-read mapping. The *KIT* region contains a high density of repetitive elements, segmental duplications and tandem repeats, all of which are poorly resolved using linear reference genomes. As a result, while the involvement of *KIT* in cattle pigmentation is evident, the precise variant underlying some breed-defining traits remains unresolved.

Recent advances in genome assembly and pangenome construction have opened new possibilities to study such complex loci. Pangenome graphs enable a more comprehensive representation of genomic diversity across individuals and breeds, allowing SV to be detected and compared within a unified framework. Using this approach, Milia et al*.* [16] recently analysed assemblies from multiple taurine cattle breeds and identified a complex structural variant ~ 66 kb upstream of *KIT*. This genomic region was associated with head depigmentation in Hereford, Simmental and other breeds. The SV consists of a repetitive element, enriched in transposable elements and variable in copy number between these two breeds (two and three copies, respectively), and was proposed to act as a regulatory element modulating *KIT* expression. Short-read data from a larger panel of animals supported the association, but for several breeds—particularly Montbéliarde and Normande—the coverage in the region was insufficient to determine the presence or absence of the variant.

Our study was built on these findings and aimed to extend the characterization of the SV upstream of *KIT* in a broader set of breeds, with a particular focus on French cattle populations. By expanding the dataset to include 13 additional breed assemblies into a local pangenome and combining long- and short-read evidence, we sought to clarify the association of this variant with the characteristic head depigmentation in several cattle breeds.

## Methods

### Genome assemblies and genomic region extraction

We analysed genome assemblies from a total of 20 cattle breeds, including 12 newly incorporated French breeds and the yak (*Bos grunniens*) (three white-headed and ten color-headed) compared to the previous study [16]. In total, 79 assemblies were considered and they are publicly available [See Additional file 1, Table S1]. These assemblies were generated using different sequencing technologies: 64 with PacBio CLR, 8 with PacBio HiFi and 1 with Oxford Nanopore (ONT).

Considering that the SV of interest has been previously reported to be located upstream of the *KIT* gene locus [16], we focused our analyses on a 2 Mb interval spanning the *KIT* region (BTA6, from 69.0 to 71.0 Mb, ARS-UCD1.2). The extraction of this genomic region data for each individual was performed through the following steps: (i) aligning the assemblies to the ARS-UCD1.2 cattle reference genome assembly using minimap2 (v2.28) [17]; (ii) identifying and extracting the target region with IMplicite Pangenome Graph (IMPG) (v0.2.1) [18], which employs all-vs-all alignments to implicitly represent the pangenome graph and project genomic intervals across multiple assemblies, using the command ‘impg-p *sample*.paf -r 6:69000000–71000000 –x’ to produce a corresponding BED file; (iii) retrieving the associated fasta sequences using SAMtools faidx (v1.20) [19].

### Local pangenome construction

The pangenome graph was generated using pggb (0.5.4) [20], based on the 2 Mb of the chromosome 6 fasta sequences previously extracted from each assembly. The parameters were adapted to the relatively small size of the targeted region, with a divergence threshold of 95.0% (based on Mash distances between assemblies, with buffer to capture locally divergent regions), a segment length of 1 kb, and a minimum match length of 31. These settings enhance match sensitivity for robust graph construction, while increasing runtime and graph complexity. To assess representation across assemblies for the 2 Mb pangenome graph, overall path coverage was calculated based on the path of each sample through all nodes using the odgi (0.8.6) [21] depth option. Coverage profiles were then visualised with BandageNG (v2022.09) [22]. This approach enables the detection of subregions with reduced coverage relative to the overall graph, which are potential hotspots of SVs. Such regions were subsequently extracted using odgi extract, and the same analytical procedure applied to the 2 Mb pangenome graph was performed on these subgraphs.

### Short read data panel and alignment on the graph

**Fig. 1:**
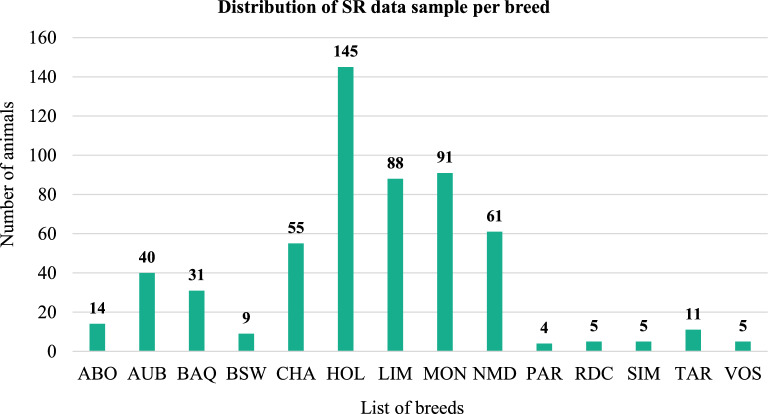
Distribution of short-read data per breed. The number of samples per breed ranged from 4 to 145

Short reads (SR) produced by Illumina Novaseq technology were considered for 564 animals corresponding to 14 cattle breeds [23] (Fig. 1). The number of individuals per breed varied from 4 (Parthenaise) to 145 (Holstein).

As the SV being studied is located in a highly repetitive region of the genome, we opted to extract, for each individual, the 30 kb sequences surrounding the *KIT* locus. To achieve this, we first aligned the raw whole genome sequences to the reference bovine genome using the bwa (0.7.17) mem algorithm [24]. From the alignment BAM file, we employed the SAMtools software (v1.20) [19] to extract the aligned sequences within the BTA6:70089519–70129,958 targeted genomic region, which we subsequently converted into a fastq format using SAMtools fastq (v1.20). The extracted reads were then aligned to the pangenome subgraph using vg giraffe (v1.51.0) [25] with the option “–named-coordinates –max-multimap = 1 -o gaf”. Finally, for each sample, node coverage was computed using gafpack (v0.1.3) [26].

## Results and discussion

### Genomic region data extraction around *KIT*

Building on the recent work of Milia et al. [16], we built a pangenome graph for a 2 Mb interval of chromosome 6 encompassing the *KIT* gene (69–71 Mb, ARS-UCD1.2). We used 79 assemblies from 20 cattle breeds, including 13 French breeds that were not represented in the previous study. To construct this local pangenome, we first extracted sequences mapping to the 2 Mb interval from all assemblies. While this region was mainly recovered with sequences from chromosome 6 only, for several samples we also observed additional alignments originating from other regions of the genome. Specifically, we detected unmapped contigs or sequences originating from chromosomes other than chromosome 6 aligning to the target interval, thereby recovering additional regions through the IMPG approach compared to only lifting over reference coordinates. To ensure that only relevant sequences were retained, we applied two filters: alignments had (i) to be at least 10 kb in length, and (ii) to cover at least 80% of the total contig length. This stringent filtering yielded 12 additional contigs (Table 1) that were subsequently incorporated into the pangenome graph.

**Table 1:** List of additional contigs included in the 2 Mb pangenome graph construction

Assembly	Breed	Additional sequence	Size	Total length of the fragment
40,509	Montbéliarde	ctg4037**	17,822	20,779
40,528	Normande	ctg1495**	26,279	26,282
40,714	Holstein	ctg1758**	17,486	17,518
41,055	Parthenaise	ctg1528**	16,319	17,675
41,185	Vosgienne	BTA29*	688,257	–
41,186	Vosgienne	ctg696**	29,216	39,067
EVO	Evolèner	BTA6*	402,022	–
YM	Yak	BTA29*	478,131	–
HN	Normande	h2tg002089c**	14,324	14,325
HN	Holstein	h1tg002669l**	37,524	37,525
HN	Holstein	h1tg002844c**	14,324	14,325
HN	Holstein	h1tg003957l**	17,871	17,872

*Sequences originating from other chromosome; **sequences originating from an unmapped contig; The term “Size” denotes the length of the segment that corresponds to the 2 Mb region surrounding the KIT locus, whereas “Total length of the fragment” indicates the complete length of the original contig from which the segment originated

In three assemblies, large fragment of the target interval mapped to chromosomes other than chromosome 6. Most notably, Vosgienne sample 41,185 and Yak haplotype assemblies, parts of the 2 Mb region also aligned to chromosome 29. Detailed inspection revealed that in the Vosgienne sample, a 688,257 bp segment of chromosome 6 (70,311,556–71,000,000 bp, based on ARS-UCD1.2 coordinates) was identified at 18,563,290–19,251,547 bp on chromosome 29. Using the UCSC liftover tool, we confirmed that this region corresponds to 6:72,886,040–73,563,656 bp on BosTaur4 (Baylor 4.0, 2007), which seems to be consistent with the Cs29 translocation previously described by Durkin et al*.* [8]. This translocation underlies the color-sidedness phenotype in cattle. For the Yak haplotype, we conducted the same analysis, and found interesting results. Based on two previous studies [8, 27], we compared the characteristics of the Cs29 translocation (see Liu et al*.* [27], Additional file 2, Fig. S1a) with our case. We observed that a portion of chromosome 6 (69,975,793–70,467,023 bp, based on ARS-UCD1.2 coordinates), containing *KIT* gene, was translocated and integrated into chromosome 29 of the Yak haplotype at coordinates 21,249,799–21,727,931 bp. Interestingly, this rearrangement showed a pattern similar to canonical Cs29 event, as the downstream part of the chromosome 6 fragment (segment DE) was repositioned at the upstream region of the insertion event on chromosome 29 (Additional file 2, Fig. S1b). This finding is consistent with the structural features described in the two previous studies. To further validate the position of this fragment, we cross-checked with the Baylor 4.0 reference assembly. In Baylor 4.0, the corresponding interval is located at 6:72,554,940–73,047,706 bp, which aligns with the region reported by Durkin et al. (72.6–73 Mb on chromosome 6). Finally, to corroborate the genomic evidence, we examined a picture of the sequenced Yak-Montbéliarde individual, which indeed displayed the color-sidedness phenotype (Additional file 2, Fig. S1c).

Altogether, these results demonstrate that a local pangenome approach not only facilitates variant discovery in complex regions but also has the capacity to reveal large-scale structural rearrangement. Although, investigating such rearrangement was not the main focus of this study, we sought to rule out the possibility that these signals simply reflected misassembles or repetitive sequences.

### Construction of a local pangenome graph and identification of a SV upstream of *KIT*

**Fig. 2:**
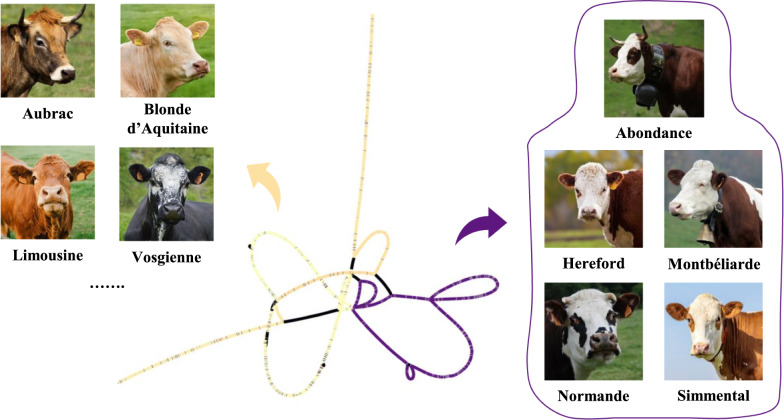
Pangenome graph of the 30 kb subregion. Yellow nodes represented the core genome, while the purple nodes were specific to the white-headed phenotype

The 2 Mb local pangenome comprised 92,558 nodes that were connected by 128,852 edges, for a total size of 2,141,415 nucleotides. Analysis of node-level coverage across all assemblies revealed a 30 kb region consisting of 1455 nodes linked by 2047 edges, which exhibited a clear distinction between white- and color-headed samples (Fig. 2). This subregion was located approximately 37.0 kb upstream of *KIT*, with the main alternative path observed around 44.4 kb upstream of the gene. Interestingly, this position is slightly closer to *KIT* than previously reported (~ 66.0 kb upstream [16]). Such differences may reflect the broader set of assemblies and the increased breed diversity considered in the present study, which could influence the resulting graph structure. They may also arise from methodological distinctions between whole-genome and local pangenome graph approaches.

Three distinct categories of nodes were observed: (i) yellow nodes were shared across all assemblies, representing the core genome; (ii) purple nodes were present in a subset of 21 assemblies and defined an alternative path of approximately 7 kb (349 nodes); (iii) black nodes corresponded to sample-specific sequences or possible artifacts from Continuous Long Read (CLR) sequencing. A more detailed examination at the purple alternative path showed that the 21 assemblies corresponded exclusively to white-headed breeds (Hereford, Abondance, Montbéliarde, Normande and Simmental; Table 2). This suggests that the same structural variant underlies head depigmentation across all white-headed breeds. Our results, therefore extend previous findings in Hereford and Simmental to additional breeds, demonstrating that Montbéliarde and Normande carry the same variant. This highlights the importance of incorporating assemblies from multiple breeds when investigating phenotypic diversity, as relying on a single reference population would have overlooked the broader distribution of this variant.

**Table 2:** Distribution of assemblies carrying the alternative path (~ 7 kb purple genomic region)

Breed	Number of assemblies in the pangenome graph	Number of assemblies showing the alternative path
Abondance*	5	3
Hereford* (*reference genome*)	1	1
Montbéliarde*	6	5
Normande*	8	7
Simmental*	6	5
Aubrac	7	0
Blonde d’Aquitaine	4	0
Brown Swiss	7	0
Charolaise	4	0
Evolèner	1	0
Highland	1	0
Holstein	9	0
Limousine	2	0
Parthenaise	3	0
Rouge Flamande	2	0
Tarentaise	5	0
Vosgienne	4	0
Yak	1	0

*White-headed breeds

However, although these five breeds contributed a total of 26 assemblies to the dataset, only 21 assemblies carried the purple path, whereas the five remaining did not. This discrepancy can be explained by the pseudo-haploid nature of CLR-based assemblies, which capture only one haplotype per individual. Thus, the absence of the variant in a given assembly does not necessarily indicate its absence in the animal, but may instead reflect allele sampling. The use of SR data helped to refine the overall presence or absence of the SV across the 14 breeds. However, fully phased long-read assemblies (*e.g.* PacBio HiFi or Oxford Nanopore) will be required to resolve this complex region, define exact breakpoints and determine copy number differences across breeds. In contrast to pseudo-haploid CLR assemblies, such phased assemblies capture both haplotypes, providing the resolution needed to characterise complex structural variations. Importantly, the incomplete representation of the variant across assemblies is consistent with the supposed dominant inheritance of the white-headed phenotype [16], where a single copy of the allele is sufficient to drive the head depigmentation trait. This further underlines the limitations of pseudo-haploid assemblies for variant discovery and highlights the need for haplotype-aware approaches.

### Validation using short-read data

**Fig. 3:**
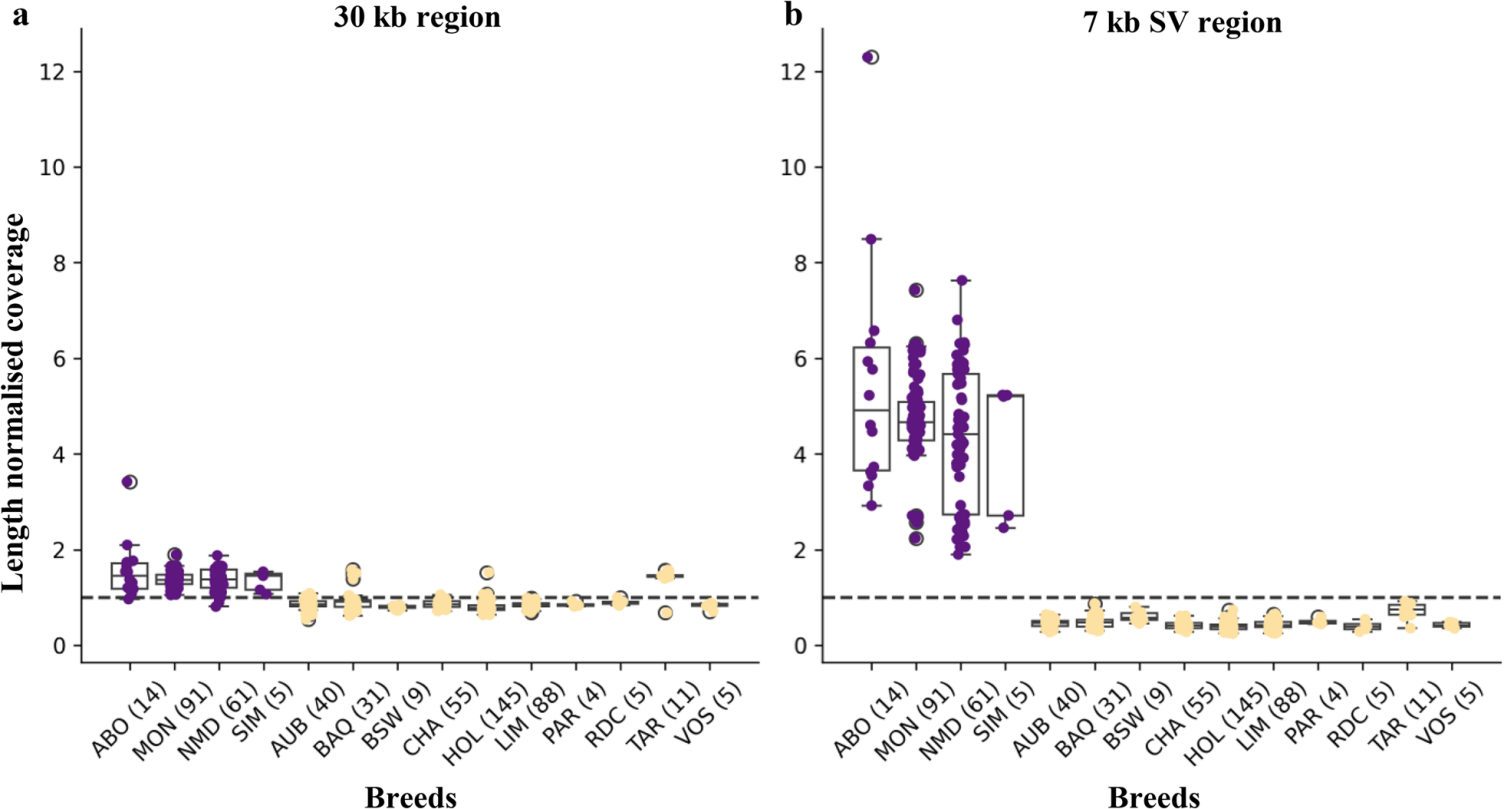
Coverage profiles for the 564 short-read samples from 14 French cattle breed. **a** Normalized coverage (over both sequencing depth and length of the region) per sample and per breed for the entire 30 kb subregion. **b** Similar to **a** but within the ~ 7 kb alternative path

Coverage profiles for the panel of 564 SR animals across the 30 kb subgraph revealed that most individuals (91%) displayed normalized coverage ranging from 0.50 to 1.50 (Fig. 3). This distribution is consistent with diploid representation. In the ~ 7 kb alternative path corresponding to the SV; however, a clear breed-specific pattern was apparent. White-headed breeds showed elevated coverage indicating the presence of duplicated sequence: 2.92–12.30 in Abondance, 2.24–7.43 in Montbéliarde, 1.90–7.63 in Normande, and 2.46–5.23 in Simmental.

In contrast, color-headed breeds exhibited coverage values close to zero, indicating that the associated sequence is missing in their assemblies. Importantly, this SR dataset also included the 64 animals for which CLR assemblies were used to build the 2 Mb pangenome graph, thereby providing a complementary layer of evidence. While CLR assemblies are pseudo-haploid and thus do not fully resolve heterozygous variants, the SR data confirm that this SV is either entirely present or entirely absent within each breed. The strong contrast between groups supports either a direct link between this SV and the white-headed phenotype or a high linkage disequilibrium with a causative variant. Moreover, its position upstream *KIT* further reinforces its implication as a causal regulatory variant underlying head depigmentation in cattle.

### Broader implications of the results

The results of this study align with previous reports showing that structural variants in or near *KIT* underlie coat color phenotypes in cattle. Similarly, in pigs [7] and horses [10], tandem duplications or transposable element insertions near this gene have been repeatedly associated with depigmentation phenotypes. This cross-species convergence highlights *KIT* as an evolutionary hotspot for regulatory variation, particularly in livestock animals where coat color has often been shaped by artificial selection.

In cattle, our findings confirm the presence of a ~ 7 kb structural variant associated with white-headed breeds, which has previously been proposed by Milia et al*.* [16] to affect regulatory elements upstream of *KIT*. This may alter the gene expression during melanocyte development and disrupt pigmentation of the head [28–31]. Short-read coverage in this region was consistently higher in white-headed breeds than in color-headed breeds. However, while Milia et al*.* [16] reported copy number differences between white-headed breeds, our data do not allow us to conclude on this point.

Together, our results demonstrate that targeted pangenome subgraph-based approaches provide an efficient and powerful framework for the detection and characterisation of structural variation at known genomic regions. This strategy offers high-resolution insights while minimizing computational cost and file size relative to whole-genome pangenome analysis. By integrating assemblies from multiple breeds with complementary short-read datasets, we were able to validate and refine previous findings, and extend the characterization of this variant to additional cattle breeds.

## Conclusions

This work illustrates how a targeted pangenome subgraph-based approach offers insights that surpass a strictly linear genome reference-based variant discovery. By extending the dataset of Milia et al*.* [16] to include 13 additional French breeds, we captured a broader range of diversity and refined the characterization of the structural variant upstream of *KIT*. Notably, our results demonstrate that the previous prediction was incomplete, as we show for the first time that both Montbéliarde and Normande cattle carry this variant—an association that could not be resolved without long-read assemblies. The combination of long-read assemblies to build pangenome graphs and public short-read datasets thus offer new opportunities to validate trait-associated variants. Beyond coat color, such approaches will contribute for uncovering structural variants underlying production, adaptation, and health traits—even when traits are not directly observable, provided that carriers and non-carriers can be distinguished—in cattle and other livestock species.

## Supplementary Information

Supplementary material 1 List of assemblies used in the study. List of assembly’s names, with sequencing technology, head coat color phenotype and data accessibility of each sample.

Supplementary material Yak chromosome 6 to chromosome 29 translocation. Illustration of the Cs29 translocation involving the *KIT* locus in the Yak haplotype. (a) Alignment of the Yak chromosome 29 segments to chromosome 6 of reference assembly ARS-UCD1.2; (b) coordinates correspondence between ARS-UCD1.2 and Baylor 4.0, used in the study of Durkin *et al.* (2012); (c) picture of the sequenced Yak-Montbéliarde crossbred individual showing the color-sidedness phenotype (source: Marie Gaborit).

## Data Availability

Accessibility to assemblies is available in supplementary file [See Additional file 1, Table S1]. Additionally, paired-end Illumina SR data (2 × 150 bp) are available for the 564 animals and publicly accessible under ENA project accession number PRJEB64023.

## References

[1] da Silva WC, da Silva ÉBR, dos Santos MRP, Camargo Junior RNC, Barbosa AVC, da Silva JAR, et al. Behavior and thermal comfort of light and dark coat dairy cows in the Eastern Amazon. Front Vet Sci. 2022;9:1006093. 36187817 10.3389/fvets.2022.1006093 PMC9516290

[2] Hansen PJ. Physiological and cellular adaptations of zebu cattle to thermal stress. Anim Reprod Sci. 2004;82–83:349–60. 15271465 10.1016/j.anireprosci.2004.04.011

[3] Cieslak M, Reissmann M, Hofreiter M, Ludwig A. Colours of domestication. Biol Rev. 2011;86:885–99. 21443614 10.1111/j.1469-185X.2011.00177.x

[4] Grichnik JM, Burch JA, Burchette J, Shea CR. The SCF/KIT pathway plays a critical role in the control of normal human melanocyte homeostasis. J Invest Dermatol. 1998;111:233–8. 9699723 10.1046/j.1523-1747.1998.00272.x

[5] PubChem. KIT - KIT proto-oncogene, receptor tyrosine kinase (human). https://pubchem.ncbi.nlm.nih.gov/gene/KIT/human. Accessed 3 Sep 2025.

[6] Nagle DL, Kozak CA, Mano H, Chapman VM, Bućan M. Physical mapping of the Tec and Gabrb1 loci reveals that the Wsh mutation on mouse chromosome 5 is associated with an inversion. Hum Mol Genet. 1995;4:2073–9. 8589683 10.1093/hmg/4.11.2073

[7] Giuffra E, Törnsten A, Marklund S, Bongcam-Rudloff E, Chardon P, Kijas JMH, et al. A large duplication associated with dominant white color in pigs originated by homologous recombination between LINE elements flanking KIT. Mamm Genome. 2002;13:569–77. 12420135 10.1007/s00335-002-2184-5

[8] Durkin K, Coppieters W, Drogemuller C, Ahariz N, Cambisano N, Druet T, et al. Serial translocation by means of circular intermediates underlies colour sidedness in cattle. Nature. 2012;482:81–4. 22297974 10.1038/nature10757

[9] Venhoranta H, Pausch H, Wysocki M, Szczerbal I, Hänninen R, Taponen J, et al. Ectopic KIT copy number variation underlies impaired migration of primordial germ cells associated with gonadal hypoplasia in cattle (Bos taurus). PLoS ONE. 2013;8:e75659. 24086604 10.1371/journal.pone.0075659 PMC3784456

[10] Dürig N, Jude R, Holl H, Brooks SA, Lafayette C, Jagannathan V, et al. Whole genome sequencing reveals a novel deletion variant in the KIT gene in horses with white spotted coat colour phenotypes. Anim Genet. 2017;48:483–5. 28444912 10.1111/age.12556

[11] Küttel L, Letko A, Häfliger IM, Signer-Hasler H, Joller S, Hirsbrunner G, et al. A complex structural variant at the KIT locus in cattle with the Pinzgauer spotting pattern. Anim Genet. 2019;50:423–9. 31294880 10.1111/age.12821

[12] Artesi M, Tamma N, Deckers M, Karim L, Coppieters W, den Van Broeke A, et al. Colour-sidedness in Gloucester cattle is associated with a complex structural variant impacting regulatory elements downstream of KIT. Anim Genet. 2020;51:461–5. 32281117 10.1111/age.12932

[13] Hayes BJ, Pryce J, Chamberlain AJ, Bowman PJ, Goddard ME. Genetic architecture of complex traits and accuracy of genomic prediction: coat colour, milk-fat percentage, and type in Holstein cattle as contrasting model traits. PLoS Genet. 2010;6:e1001139. 20927186 10.1371/journal.pgen.1001139 PMC2944788

[14] Qanbari S, Pausch H, Jansen S, Somel M, Strom TM, Fries R, et al. Classic selective sweeps revealed by massive sequencing in cattle. PLoS Genet. 2014;10:e1004148. 24586189 10.1371/journal.pgen.1004148 PMC3937232

[15] Mészáros G, Petautschnig E, Schwarzenbacher H, Sölkner J. Genomic regions influencing coat color saturation and facial markings in Fleckvieh cattle. Anim Genet. 2015;46:65–8. 25515556 10.1111/age.12249

[16] Milia S, Leonard AS, Mapel XM, Bernal Ulloa SM, Drögemüller C, Pausch H. Taurine pangenome uncovers a segmental duplication upstream of KIT associated with depigmentation in white-headed cattle. Genome Res. 2025;35:1041–52. 39694857 10.1101/gr.279064.124 PMC12047182

[17] Li H. Minimap2: pairwise alignment for nucleotide sequences. Bioinformatics. 2018;34:3094–100. 29750242 10.1093/bioinformatics/bty191 PMC6137996

[18] impg: implicit pangenome graph; 2025. https://github.com/pangenome/impg. Accessed 3 Sep 2025.

[19] Danecek P, Bonfield JK, Liddle J, Marshall J, Ohan V, Pollard MO, et al. Twelve years of SAMtools and BCFtools. Gigascience. 2021;10:giab008. 33590861 10.1093/gigascience/giab008 PMC7931819

[20] Garrison E, Guarracino A, Heumos S, Villani F, Bao Z, Tattini L, et al. Building pangenome graphs. Nat Methods. 2024;21:2008–12. 39433878 10.1038/s41592-024-02430-3

[21] Guarracino A, Heumos S, Nahnsen S, Prins P, Garrison E. ODGI: understanding pangenome graphs. Bioinformatics. 2022;38:3319–26. 35552372 10.1093/bioinformatics/btac308 PMC9237687

[22] Korobeynikov A. BandageNG. 2025. https://github.com/asl/BandageNG. Accessed 3 Sep 2025.

[23] Boussaha M, Eché C, Klopp C, Grohs C, Milhes M, Suin A, et al. Whole genome short read data from 567 bulls of 14 breeds provides insight into genetic diversity of French cattle. Data Brief. 2025. 10.1016/j.dib.2025.112049. 41036251 10.1016/j.dib.2025.112049 PMC12481132

[24] Li H. Aligning sequence reads, clone sequences and assembly contigs with BWA-MEM. arXiv:1303.3997v2 [q-bio.GN]; 2013. Accessed 2 Sep 2025.

[25] Sirén J, Monlong J, Chang X, Novak AM, Eizenga JM, Markello C, et al. Pangenomics enables genotyping of known structural variants in 5202 diverse genomes. Science. 2021;374:abg8871. 34914532 10.1126/science.abg8871 PMC9365333

[26] Pangenome/gafpack; 2024. https://github.com/pangenome/gafpack. Accessed 3 Sep 2025.

[27] Liu X, Liu W, Lenstra JA, Zheng Z, Wu X, Yang J, et al. Evolutionary origin of genomic structural variations in domestic yaks. Nat Commun. 2023;14:5617. 37726270 10.1038/s41467-023-41220-x PMC10509194

[28] Berrozpe G, Timokhina I, Yukl S, Tajima Y, Ono M, Zelenetz AD, et al. The Wsh, W57, and Ph Kit expression mutations define tissue-specific control elements located between −23 and −154 kb upstream of Kit. Blood. 1999;94:2658–66. 10515869

[29] Berrozpe G, Agosti V, Tucker C, Blanpain C, Manova K, Besmer P. A distant upstream locus control region is critical for expression of the Kit receptor gene in mast cells. Mol Cell Biol. 2006;26:5850–60. 16847336 10.1128/MCB.01854-05 PMC1592758

[30] Garrido MC, Bastian BC. Kit as a therapeutic target in melanoma. J Invest Dermatol. 2010;130:20–7. 19847190 10.1038/jid.2009.334 PMC2831053

[31] Hu S, Chen Y, Zhao B, Yang N, Chen S, Shen J, et al. Kit is involved in melanocyte proliferation, apoptosis and melanogenesis in the Rex Rabbit. PeerJ. 2020;8:e9402. 32596061 10.7717/peerj.9402 PMC7306216

