# Supplementary figures and images for: Pangenome-based association testing between a structural variant located upstream of the KIT gene and head depigmentation across a diverse panel of cattle breeds
Source: Genet Sel Evol. 2026 Feb 26;58:17. doi: 10.1186/s12711-026-01037-w (PMC12964817; doi:10.1186/s12711-026-01037-w)

**a**

**ARS-UCD1.2**  
Chromosome 6

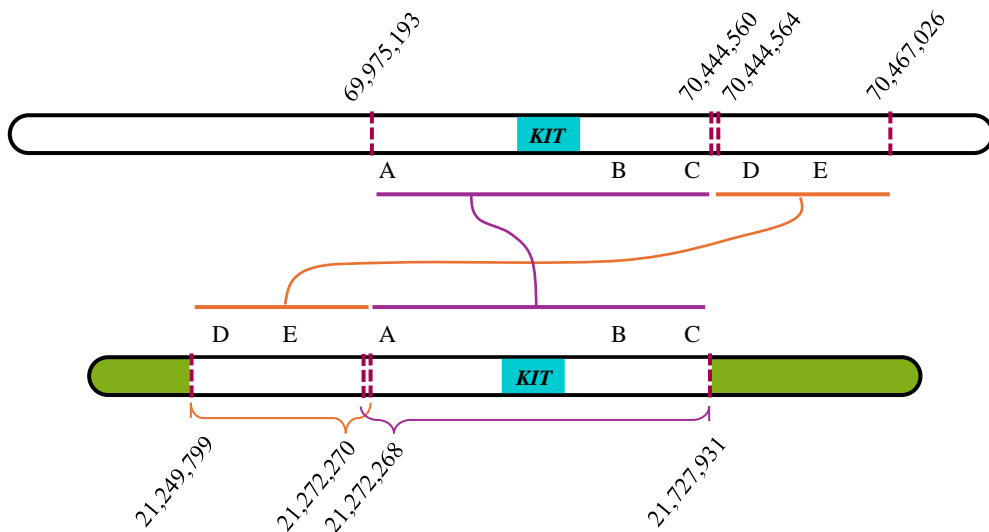

**b**

**ARS-UCD1.2**  
Chromosome 6

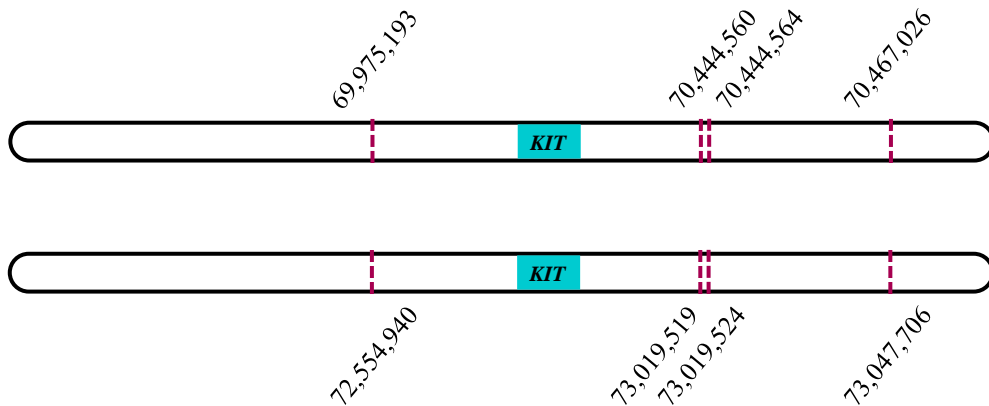

**c**

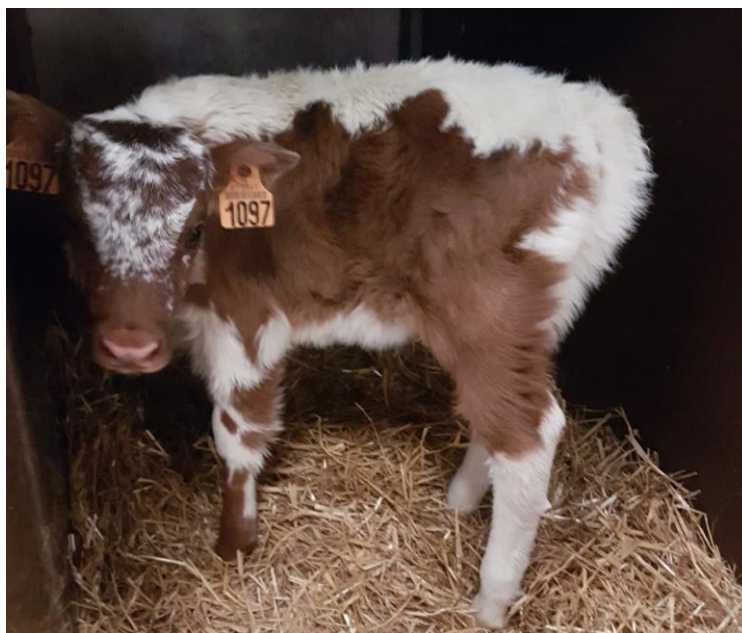

Supplement: Supplementary file 2 — Supplementary material Yak chromosome 6 to chromosome 29 translocation. Illustration of the Cs29 translocation involving the KIT locus in the Yak haplotype. (a) Alignment of the Yak chromosome 29 segments to chromosome 6 of reference assembly ARS-UCD1.2; (b) coordinates correspondence between ARS-UCD1.2 and Baylor 4.0, used in the study of Durkin et al. (2012); (c) picture of the sequenced Yak-Montbéliarde crossbred individual showing the color-sidedness phenotype (source: Marie Gaborit). [file 12711_2026_1037_MOESM2_ESM.pdf]
